# Biological age acceleration associates with Alzheimer's disease plasma biomarker levels

**DOI:** 10.1002/alz.71005

**Published:** 2026-01-22

**Authors:** Jaclyn M. Eissman, Yiyi Ma, Min Qiao, Dolly Reyes‐Dumeyer, Angel Piriz, Annie J. Lee, Rafael A. Lantigua, Martin Medrano, Diones Rivera Mejia, Lawrence S. Honig, Francine Grodstein, David A. Bennett, Philip L. De Jager, Clifton L. Dalgard, Richard Mayeux, Badri N. Vardarajan

**Affiliations:** ^1^ The Taub Institute for Research on Alzheimer's Disease and the Aging Brain Columbia University New York New York USA; ^2^ Gertrude H. Sergievsky Center Columbia University New York New York USA; ^3^ Vagelos College of Physicians and Surgeons Columbia University New York New York USA; ^4^ Pontificia Universidad Católica Madre y Maestra Santiago República Dominicana; ^5^ CEDIMAT Santo Domingo República Dominicana; ^6^ Universidad Nacional Pedro Henriquez Ureña Santo Domingo República Dominicana; ^7^ Rush Alzheimer's Disease Center Rush University Medical Center Chicago Illinois USA; ^8^ Center for Translational & Computational Neuroimmunology Columbia University New York New York USA; ^9^ Department of Anatomy, Physiology & Genetics Uniformed Services University of the Health Sciences Bethesda Maryland USA; ^10^ The American Genome Center, Center for Military Precision Health Uniformed Services University of the Health Sciences Bethesda Maryland USA

**Keywords:** age acceleration, Alzheimer's disease, biological aging, epigenetic clocks, methylation, plasma biomarkers, preclinical disease, risk stratification

## Abstract

**INTRODUCTION:**

Epigenetic clocks associate with neuropathology and Alzheimer's disease (AD) clinical risk, but findings are mixed regarding whether clocks associate with blood‐based biomarkers and in non‐European populations.

**METHODS:**

We calculated biological age and age acceleration from blood methylation data in 704 older Hispanic adults and tested associations with clinical diagnosis and *antemortem* biomarker levels.

**RESULTS:**

Age acceleration was significantly associated with sex, clinical diagnosis, and levels of eight plasma biomarkers, including P‐tau217 levels. Additionally, biomarker associations trended more significantly among *APOE*‐ε4 non‐carriers. We also identified that methylation levels in CD4 and CD8 T‐cell types are associated with age acceleration.

**DISCUSSION:**

We demonstrated that biological age acceleration, measured in blood, in a Hispanic cohort enriched for preclinical individuals, can stratify clinical AD risk and is associated with plasma AD biomarker levels.

**Highlights:**

1. Blood‐based aging clocks associate with Alzheimer's disease plasma biomarker levels.
2. Biological aging appears relevant to pathological aging in apolipoprotein E (*APOE)* ‐ε4 non‐carriers.
3. Immune T‐cell composition relates to biological aging.

## 1 BACKGROUND

Alzheimer's disease (AD) is a public health crisis affecting 6.9 million Americans, and this number is expected to nearly double in the next 35 years.^1^ Advanced age increases the odds of developing AD, increasing odds two‐fold in every 10‐year span beginning at age 65.^1^ However, there is a non‐linear relationship between chronological age and AD risk, and there is much heterogeneity in aging trajectories over time. Up to 30%–50% of adults who receive a neuropathological classification of AD at autopsy were cognitively unimpaired throughout life,^2^ demonstrating the need for novel biomarkers for early risk detection and stratification.

DNA methylation (DNAm) signatures mirror aging and disease at a biomolecular level,^3^ including both a global hypomethylation phenomenon and hypermethylation at typically unmethylated regions, CpG islands, often found near gene promoters.^4^ Studies have found changes at specific CpG sites that change with respect to aging, which can be leveraged to calculate an individual's “biological age”.^5^ Brain DNAm changes have associated with AD neuropathology,^6^, ^7^ including in preclinical AD,^6^ and accelerated epigenetic aging has correlated with progression to mild cognitive impairment (MCI) and AD as well as with cognitive decline.^8^ Epigenetic clocks can accurately predict those that will go on to develop dementia^9^, ^10^ up to 3 years prior to clinical diagnosis.^10^ These studies support the vitality of epigenetic clocks as a potential early‐stage AD biomarker.

Over the last decade, several epigenetic clocks have been developed and validated, including Horvath,^5^ Hannum,^11^ and Levine (PhenoAge)^12^ clocks, which each capture different aspects of aging. Additionally, cortical clocks^13^, ^14^ were developed using *postmortem* brain methylation levels from the Religious Orders Study and Rush Memory and Aging Project (ROS/MAP).^13^, ^14^ One limitation of many epigenetic clock studies is that training was exclusive to non‐Hispanic whites, limiting generalizability to more diverse groups. A recent study demonstrated that epigenetic clocks show an attenuated correlation of chronological and biological age in individuals of non‐European descent.^15^ A second limitation is that, to our knowledge, biological aging associations with AD neuropathologic burden have primarily been established with *postmortem* measures.^7^, ^13^, ^16^ While the association of biological age strongly correlates with *postmortem* AD pathology, it needs to be established whether epigenetic clocks can predict *antemortem* AD risk and neuropathological burden.

Thus, in this study, we expanded on published work by calculating biological age and age acceleration in blood with two well‐established aging clocks (Horvath and Hannum) in an aging Hispanic cohort enriched for preclinical AD. We evaluated the degree of *antemortem* AD risk stratification by testing the association of biological age and age acceleration (in blood) with a panel of plasma AD biomarkers. We additionally examined associations stratified by sex and by apolipoprotein E (*APOE)* ‐ε4 carrier status to have a more complete understanding of the association of biological aging with AD risk.

## 2 METHODS

### 2.1 Study participants

In this analysis, we leveraged Estudio Familiar de Influencia Genetica en Alzheimer (EFIGA),^17^ which is a cohort study comprised of older individuals with AD or aged‐matched healthy controls with a family history of AD. All participants were of Caribbean Hispanic descent. Recruitment for EFIGA began in 1998 and took place in Washington Heights, New York, and the Dominican Republic, and participants were followed every 2 years. Participants had a family history interview, received an AD evaluation, a neuropsychological test battery, and medical and neurological exams. Additionally, NINCDS‐ADRDA criteria^18^, ^19^ were applied to determine if participants were cognitively unimpaired or had a probable or possible AD diagnosis, and the Clinical Dementia Rating^20^, ^21^, ^22^ was also used to help determine the severity of disease. For this analysis, we created a binarized clinical diagnosis variable, whereby an individual was either classified as cognitively normal or clinical AD. Please note, in this analysis, we leveraged an unrelated set of individuals from EFIGA.

**RESEARCH IN CONTEXT**

1. **Systematic review**: Previous studies established that biological aging, quantified by epigenetic clocks, associates with *postmortem* Alzheimer's disease (AD) neuropathology burden. Points of clarification remaining include if biological aging associates with (1) *antemortem* AD neuropathology levels, (2) in diverse populations, (3) in apolipoprotein E *(APOE)*‐ε4 carriers and non‐carriers alike. We sought to address these questions.
2. **Interpretation**: Our findings demonstrated that biological age and age acceleration are associated with AD plasma biomarker levels and do so more strongly among *APOE*‐ε4 non‐carriers; we validated our findings with *postmortem* measures. Additionally, we showed that T‐cell composition is important in biological aging.
3. **Future directions**: Future work should focus on site‐specific methylation changes relevant to AD pathogenesis among *APOE*‐ε4 non‐carriers. In addition, future studies should further evaluate the role of T‐cell remodeling with respect to biological aging.

### 2.2 Plasma biomarkers data generation

Blood for plasma was collected in dipotassium ethylenediaminetetraacetic acid tubes, and 2000 g were centrifuged for 15 minutes at 4°C. The spun down blood was next aliquoted to polypropylene tubes, frozen, and stored at −80°C. Assays with specificity to each biomarker measured were applied to aliquots with the Simoa HD‐X platform (Quanterix).^23^ Plasma biomarkers collected included Aβ40, Aβ42, total‐tau, GFAP, NfL, phosphorylated tau (P‐tau) 181, 217, and 231, and we additionally calculated an Aβ42/Aβ40 ratio. For more details on plasma biomarker data collection, please see our published protocol.^23^ Prior to analysis, we removed values of biomarker levels that were more than five standard deviations above or below the mean across samples.

### 2.3 Plasma epigenetic data generation

Blood was collected in PAXgene tubes at the same time as when the plasma biomarkers were collected, as described above. To identify 5‐methylcytosine and 5‐hydroxymethylcytosine, we applied the NEBNext Enzymatic Methyl‐Seq workflow^24^ with the following steps. First, 10 ng of DNA was treated with TET2 oxidation of 5‐methylcytosine, and second, modified cystines were deaminated with APOBEC. Next, polymerase chain reaction (PCR) amplification was conducted with NEB Unique dual index primer pairs, and an Illumina NovaSeq 6000 sequencer was used for sequencing. Once sequenced, FASTQ files were aligned (GRCh38) leveraging the Bismark (v. 0.24.2)^25^ software package. Finally, duplicate reads that had MAPQ scores < 20 were subsequently filtered out. We additionally performed a deconvolution step on the cleaned whole‐genome (plasma) methylation data with the EpiDISH^26^ package in R. Our deconvoluted data contained the following cell‐type proportions for which we had reliable markers: CD4 and CD8 memory T‐cells, naïve CD4 and CD8 T‐cells, regulatory T‐cells, natural killer cells, naïve and memory B‐cells, eosinophils, basophils, monocytes, and neutrophils.

### 2.4 Statistical analyses

#### 2.4.1 Calculating epigenetic clocks

We calculated epigenetic clock ages (i.e., biological age) with the “methylclock” R package^27^ that is publicly available on GitHub (https://github.com/isglobal‐brge/methylclock). To prepare for clock calculations, we first matched chromosome and base pair positions from our methylation data with the Illumina Array Infinium Methylation EPIC (v. 2.0) CpG Product File annotations (GRCh38). This was necessary as the clocks each used Illumina array CpG site nomenclature. Before clock calculations, we filtered out a sample or a CpG site if it had > 20% missing data. We calculated two clocks with the *DNAmAge* function (in the methylclock package), which allowed for calculation of the Horvath^5^ and Hannum clocks,^11^ both of which leverage elastic net modeling. Any missing data at a required CpG site were imputed during clock age calculations, which, by default, of the methylclock package, implements the *impute.knn* function (impute R package), applying the K‐nearest neighbor method for imputation.^27^ In addition to calculating biological age, we calculated age acceleration (with the methylclock package), which is defined by the residual of biological age regressed onto chronological age.

#### 2.4.2 Global ancestry proportion calculations

Previous studies have reported differential methylation rates by ethnicity,^28^ but their impact on biological aging clocks is not clear. We computed global proportions of three continental ancestries–European, African, and Amerindian–in Caribbean Hispanic participants using the ADMIXTURE package^29^ (v 1.3.0). Of the 704 EFIGA samples included in this analysis, we had whole‐genome sequencing (WGS) data^30^ for *N* = 588. We used genome‐wide common single‐nucleotide polymorphisms (SNPs) with minor allele frequency > 5% and reference genetic data from individuals of the three continental ancestries from the Human Genome Diversity Project (HGDP^31^; https://www.internationalgenome.org/data‐portal/data‐collection/hgdp). We used European (CEU), African (YRI), and Amerindian (AMR: PEL, PUR, MXL, CLM subpopulations combined) reference populations and ran a supervised model for global ancestry assessment (ADMIXTURE package). This resulted in three ancestry proportions per sample: %CEU, %YRI, %AMR.

#### 2.4.3 Association testing with biological age

All association testing was conducted in R with linear models for quantitative outcomes and generalized linear models for binary outcomes. Biological age and age acceleration from each clock served as predictors for models; models were adjusted for age at blood draw. If quantitative predictors and outcomes were not already scaled, we scaled variables in the model to get standardized output. All models were adjusted for self‐reported sex. Secondary analyses included an identical set of models stratified first by sex (note–these models did not covary for sex) and then by *APOE*‐ε4 carrier status (defined by presence of 1‐2 ε4 alleles vs. no ε4 alleles). Within each set of outcomes, the false‐discovery rate (FDR) procedure was used to adjust *p*‐values within each analysis, with significance set at FDR < 0.05. FDR‐correction was performed separately per clock, and we considered an association significant if an outcome survived FDR‐correction in one or both clocks.

#### 2.4.4 Association testing with age acceleration

Initially, we tested the effect of biological age without adjustment of chronological age to avoid collinearity in the regression models. However, since biological and chronological ages are highly correlated, we tested the association of AD biomarkers with the residual effect of biological age after regressing out the effects of chronological age (i.e., age acceleration). This allowed us to identify effects of biological aging beyond chronological age.

#### 2.4.5 Sensitivity analyses addressing genetic context

To address the fact that the EFIGA cohort is genetically admixed of mainly European, African, and Amerindian ancestry, we conducted sensitivity analyses adjusting for global ancestry proportions. For the main statistical models in all individuals, we repeated the analyses as in Sections 2.4.3 and 2.4.4 with the addition of adjusting for %YRI and % AMR ancestry (%CEU was used as the reference).

#### 2.4.6 Validation cohort overview and methylation data preprocessing

We sought to validate our findings in *postmortem* brain epigenetic data and *postmortem* measures of neuropathology. To conduct validation analyses, we leveraged the ROS/MAP cohort.^32^ ROS and MAP are both longitudinal clinical–pathological studies that followed participants from the Chicago area over time, whereby ROS recruited nuns, priests, and brothers from religious orders, and MAP recruited community‐dwelling participants from retirement and senior living areas. ROS/MAP participants enroll without known dementia and agree to annual neurological exams and cognitive performance tests, and brain donation upon death. Genome‐wide methylation levels (dorsolateral prefrontal cortex)^33^ were processed with the Illumina Infinium HumanMethylation450 BeadChip.^6^ Probe‐based quality control steps included (1) retaining high‐quality probes determined based on a detection *p* < 0.01 (across samples),^33^ (2) dropping probes that cross‐hybridized with sex chromosomes,^35^, ^36^ and (3) removing probes that overlapped with variants (± 10 bp) with a minor allele frequency (MAF) ≥0.01.^33^ Sample‐based quality control steps included (1) removing samples that fell outside of three standard deviations of the mean from the first three principal components generated from a principal component analysis of 50,000 randomly selected probes,^33^ and (2) removing samples with low bisulfite conversion efficiency.^33^

#### 2.4.7 Validation association testing in ROS/MAP

In ROS/MAP, we calculated biological age and age acceleration (in brain) leveraging identical methods as described above; models were adjusted for age at death. Further, to maximize sample size, we retained samples of all ancestry groups; although to note, 95% of the ROS/MAP samples have genetically determined European ancestry. We tested biological age and age acceleration associations with measures of *postmortem* neuropathology,^37^ including four measures: the square root of the mean for eight quantified measures of (1) cortical amyloid plaques (i.e., amyloid; quantified by immunohistochemistry) and (2) neurofibrillary tau tangle density (i.e., tangles; AT8, immunohistochemistry), (3) mean count of tau tangles divided by standard deviation of five silver‐stained brain regions (neurofibrillary tangle [NFT]), as well as (4) a quantitative summary score for neuritic and diffuse plaques and NFTs across five brain regions (global pathology). We also created a binarized clinical diagnosis variable, whereby individuals with no cognitive impairment or mild cognitive impairment were in one group, and those with clinical AD were in another group.

### 2.5 Data availability

All results are included in the main text or the supplementary materials of the manuscript. The raw plasma biomarker and whole‐genome methylation data will be shared with qualified investigators using the request form available here: https://cumc.co1.qualtrics.com/jfe/form/SV_dmck0uV3A91pmzb. ROS/MAP resources can be requested at https://www.radc.rush.edu and www.synpase.org.

## 3 RESULTS

**FIGURE 1:**
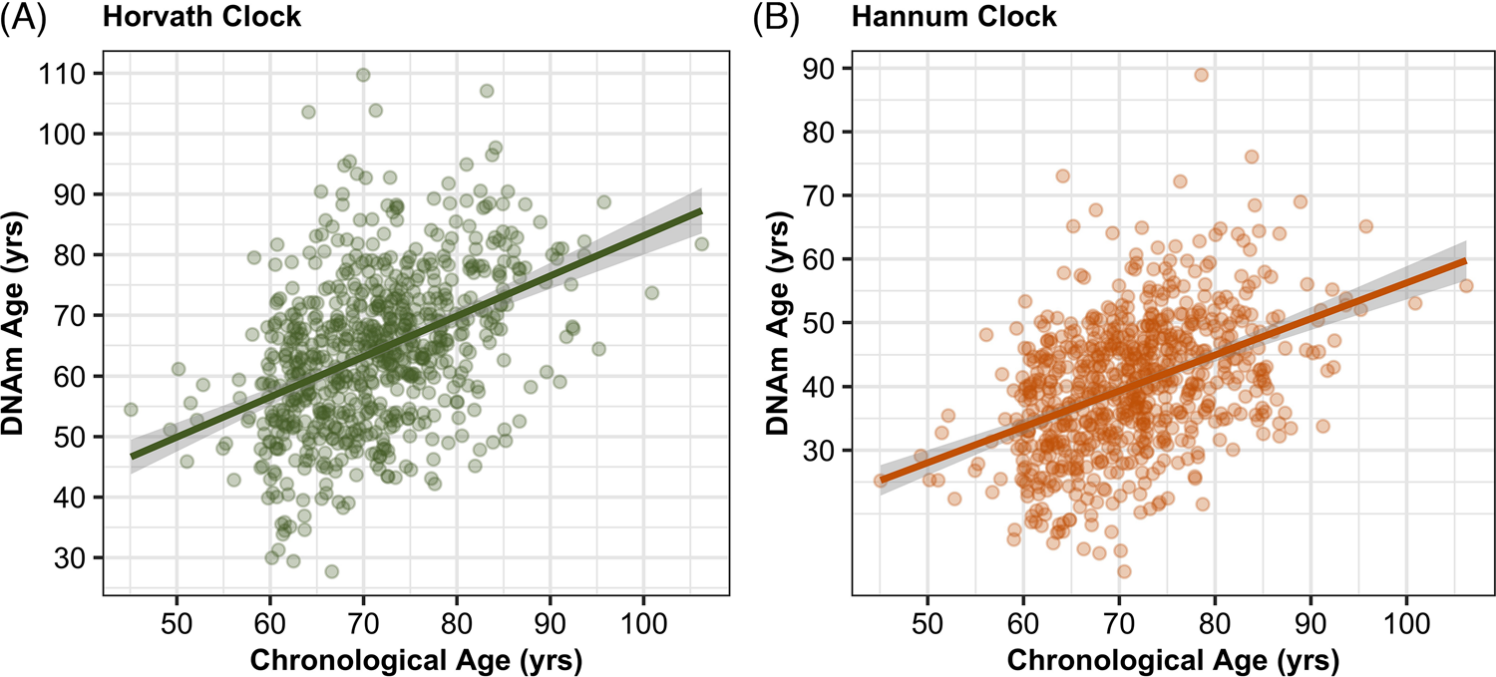
Biological age is correlated with chronological age. Biological age (DNAm) derived from (A) Horvath and (B) Hannum blood‐based epigenetic clocks of aging are correlated with chronological age in a Hispanic cohort enriched for individuals with preclinical Alzheimer's disease.

**FIGURE 2:**
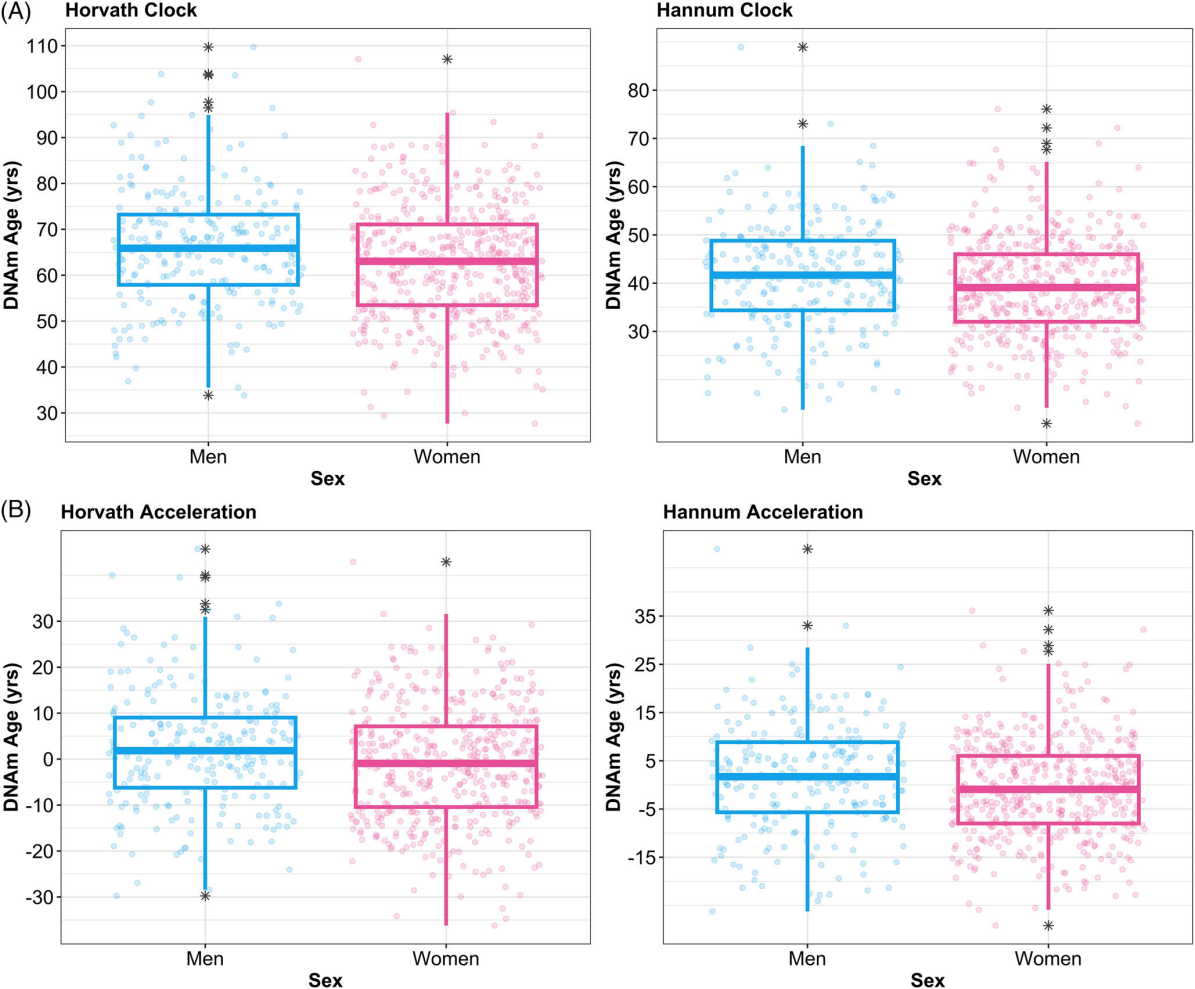
Biological aging is significantly associated with sex. Both biological age [DNAm; (A)] and age acceleration (B) derived from epigenetic clocks are significantly associated with sex. (A, B) Men have both a slightly older biological age and a slightly faster biological age acceleration as compared to women.

**FIGURE 3:**
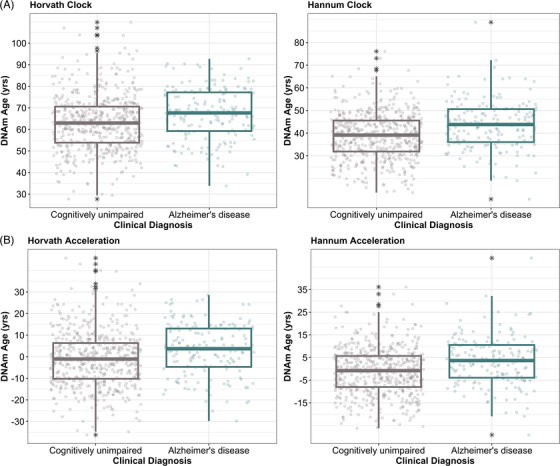
Biological aging is significantly associated with clinical diagnosis. Biological age [DNAm; (A)] derived from epigenetic clocks is not associated with clinical diagnosis, but age acceleration (B) is significantly associated. While those with a clinical Alzheimer's disease diagnosis do not have a statistically significant older biological age than clinical controls (A), clinical cases do have a faster age acceleration as compared to their age‐matched healthy counterparts (B).

**FIGURE 4:**
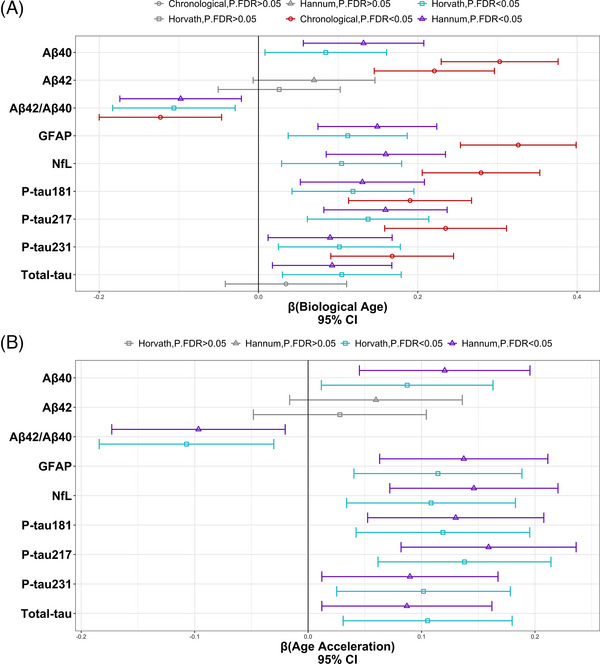
Biological age and age acceleration are significantly associated with Alzheimer's disease plasma biomarker levels. (A) Biological age and (B) age acceleration, both derived from epigenetic clocks, are significantly associated with eight out of nine plasma biomarkers tested, with each biomarker name listed on the *y*‐axis. (A, B) The *x*‐axis on both panels shows a 95% confidence interval calculated from the beta and standard error from each model. Age type is denoted by the shape and color of each plotted beta point estimate, with all nonsignificant results in gray.

**FIGURE 5:**
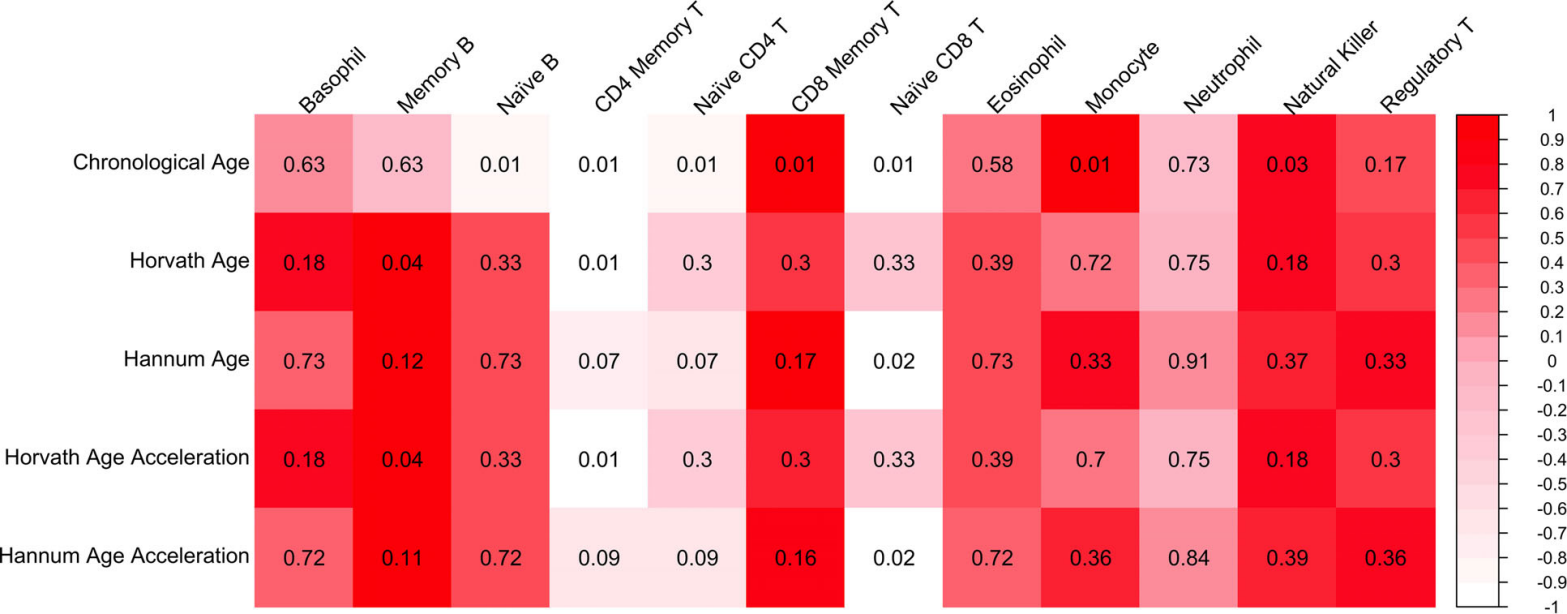
Biological aging is significantly associated with immune cell‐type proportions. Red, white, and pink coloring represents the strength of effect size, represented by beta values (rescaled in the figure from −1 to 1 for visualization purposes) from each model, and the black labeled values in each box represent the false‐discovery‐rate‐adjusted *p*‐values from each model. Chronological aging is associated with multiple immune cell types (first row). Both biological age (second and third rows) and age acceleration (fourth and fifth rows) derived from epigenetic clocks show that memory CD4 T‐cells, naïve CD8 T‐cells, and memory B‐cells are significantly associated with both biological age and age acceleration.

Participants' characteristics are outlined in Table 1. All findings from the following results sections are presented in Figures 1, 2, 3, 4, 5 and in Tables S1–S18.

**TABLE 1:** EFIGA participant overview.

Parameter	Healthy controls (*N* = 534)	Clinical AD cases (*N* = 169)	Full sample (*N* = 704)
Age in years *mean(range)*			
Chronological	69.39 (45.08–95.77)	76.91 (55.26–106.23)	71.20 (45.08–106.23)
Biological, Horvath	62.97 (27.68–109.72)	67.27 (33.84–92.75)	63.99 (27.68–109.72)
Acceleration, Horvath	−1.04 (−36.21–45.71)	3.35 (−29.77–28.54)	6.16 × 10^−16^ (−36.21–45.71)
Biological, Hannum)	38.83 (13.78–76.08)	43.76 (10.91–88.93)	40.02 (10.91–88.93)
Acceleration, Hannum)	−1.16 (−26.23–36.14)	3.58 (−29.15–48.90)	4.14 × 10^−16^ (−29.15–48.90)
Sex *(N)%*			
Men	176 (32.96%)	62 (36.69%)	238 (33.81%)
Women	358 (67.04%)	107 (63.31%)	465 (66.05%)
Plasma biomarker levels *mean(range)*			
P‐tau181	2.18 (0.16–8.19)	3.01 (0.70–8.36)	2.38 (0.16–8.36)
P‐tau217	0.36 (0.01–1.48)	0.63 (0.04–2.24)	0.43 (0.01–2.24)
Aβ42/Aβ40	0.05 (0.003–0.12)	0.05 (0.02–0.09)	0.05 (0.003–0.12)
*APOE* ε4 carrier status *N*(%)			
Non‐carrier	343 (64.23%)	96 (56.80%)	439 (62.36%)
Carrier (≥1 allele)	182 (34.08%)	72 (42.60%)	254 (36.08%)

*Note*: percentages are based on total sample sizes of full sample, healthy controls, and clinical AD cases, and thus, numbers may not add up to 100% if any individuals are missing information; plasma biomarker statistics do not include individuals ± 5 standard deviations from the mean values across samples.

Abbreviations: AD, Alzheimer's disease; APOE, apolipoprotein E; EFIGA, Estudio Familiar de Influencia Genetica en Alzheimer.

### 3.1 Overview of study participants

We leveraged 704 individuals from the EFIGA cohort study, including 169 clinical AD cases and 534 age‐matched healthy controls (Table 1); all individuals were of Caribbean Hispanic descent. Individuals with clinical AD had an older chronological age (*t* = −10.64, *p* = 3.87 × 10^−22^) as compared to healthy controls and the full sample. Our cohort included more women (65.05%) than men (33.81%) and contained 36.08% *APOE*‐ε4 carriers (1‐2 ε4 alleles) and 62.36% ε4 non‐carriers (no ε4 alleles). Additionally, in Table 1, we present biomarker averages and ranges among all individuals, healthy controls, and those with clinical AD. As expected, plasma P‐tau 181 (*t* = −6.13, *p* = 4.76 × 10^−9^) and 217 (*t* = −6.49, *p* = 8.69 × 10^−10^) levels were higher in AD as compared to both healthy controls and the full sample. A clinical‐pathological cohort of aging and AD, ROS/MAP, was leveraged for validation analyses; please see Methods for more details on ROS/MAP.

### 3.2 Biological age is correlated with chronological age

We first evaluated the correlation of chronological age with biological age. As expected, chronological age (i.e., age at blood draw) was correlated with biological age (Figure 1), for both the Horvath clock (Pearson *R* ^2 ^= 0.43; Figure 1A) and the Hannum clock (Pearson *R* ^2 ^= 0.43; Figure 1B). We observed similar correlations in the ROS/MAP brain epigenetic data, which confirmed strong correlations with chronological age (i.e., age at death) for both the Horvath (Pearson *R* ^2 ^= 0.70) and Hannum (Pearson *R* ^2 ^= 0.59) clocks. We note that our findings align with a recent study^15^ showing partially attenuated correlations in Hispanic datasets (i.e., EFIGA), and higher correlations in European datasets (i.e., ROS/MAP).

### 3.3 Biological aging is associated with sex

We tested the association of biological age (adjusted for chronological age) and age acceleration with sex, and observed that sex was significantly associated with biological age (Figure 2A), for both the Horvath clock (*β* = 0.19, *p* = 8.44 × 10^−3^) and the Hannum clock (*β* = 0.15, *p* = 3.58 × 10^−2^). We confirmed the association, in ROS/MAP brain epigenetic data, of biological age with sex for the Horvath clock (*β* = 0.12, *p* = 3.49 × 10^−2^), but the Hannum clock was just below statistical significance (*β* = 0.10, *p* = 0.11). Age acceleration calculated from both the Horvath clock (*β* = −0.25, *p* = 1.54 × 10^−3^) and the Hannum clock (*β* = 0.22, *p* = 5.30 × 10^−3^) was significantly associated with sex (Figure 2B) in Hispanics. We additionally validated these associations of age acceleration with sex in ROS/MAP for both the Horvath clock (*β* = −0.23, *p* = 2.93 × 10^−3^) and the Hannum clock (*β* = −0.19, *p* = 1.40 × 10^−2^). Overall, on average, men had both a slightly older biological age (Figure 2A) and a slightly faster age acceleration (Figure 2B), the latter suggesting that when effects of chronological age are regressed out, biological aging is modestly more rapid in men compared to women.

### 3.4 Age acceleration is associated with clinical diagnosis

We tested the association of biological age (adjusted for chronological age) and age acceleration with a clinical diagnosis of AD (Figure 3), adjusted for sex. Clinical diagnosis (Horvath: *β* = −0.07, *p* = 0.45; Hannum: *β* = 0.07, *p* = 0.44) was not significantly associated with biological age for either clock (Figure 3A), and likewise in ROS/MAP biological age with not associated with clinical diagnosis in the Horvath clock (*β* = 0.08, *p* = 0.20) but was associated in the Hannum clock (*β* = 0.19, *p* = 4.88 × 10^−3^). However, clinical diagnosis (Horvath: *β* = 0.34, *p* = 1.29 × 10^−4^; Hannum: *β* = 0.44, *p* = 5.54 × 10^−7^) was associated (in EFIGA) with age acceleration derived from both clocks (Figure 3B), and we additionally validated these associations with age acceleration in ROS/MAP (Horvath: *β* = 0.51, *p* = 5.97 × 10^−11^; Hannum: *β* = 0.55, *p* = 2.18 × 10^−12^). Overall, clinical cases and controls tended to have a similar biological age, but when the effects of chronological age were regressed out, it became apparent that biological aging was more rapid in clinical AD cases compared to controls (Figure 3B).

### 3.5 Biological aging is associated with *antemortem* AD biomarkers

#### 3.5.1 Biological age

We tested the association of biological age with a panel of AD plasma biomarkers (Figure 4A and Tables S1–S3) and considered associations statistically significant if at least one clock association survived adjustment for multiple comparisons (FDR < 0.05). Of the nine biomarkers tested, eight were significant in at least one clock (Figure 4A and Table S1), including P‐tau measures 181, 217, and 231, as well as Aβ42/Aβ40, Aβ40, glial fibrillary acidic protein (GFAP), total‐tau, and neurofilament light chain (NfL), whereas Aβ42 levels were not associated with either clock. To note, of the biomarker associations, total‐tau was significantly associated with biological age (Horvath: *β* = 0.11, *p* = 6.13 × 10^−3^, p.FDR = 1.05 × 10^−2^; Hannum: *β* = 9.25 × 10^−2^, *p* = 1.58 × 10^−2^, p.FDR = 2.04 × 10^−2^) and not with chronological age (*β* = 3.45 × 10^−2^, *p* = 3.75 × 10^−1^, p.FDR = 3.75 × 10^−1^). We validated biological age associations in ROS/MAP,^37^ leveraging *postmortem* measures of amyloid, tau tangle density, silver‐stained tau burden (NFT), and a global pathology summary score (please see Methods for more information on *postmortem* measures). In ROS/MAP, all four measures were significantly associated with biological age (Tables S4–S6). Notably, we observed a trend (in Hispanics) that biological age associations with biomarkers were more significantly associated among *APOE*‐ε4 non‐carriers as compared to ε4 carriers (Table S3), and we likewise observed this pattern in ROS/MAP with the four autopsy measures of neuropathology (Table S6; please note, effect estimates in ROS/MAP are stronger than previously reported,^13^, ^16^ possibly because we tested the effects of biological age and chronological age in separate models.)

#### 3.5.2 Age acceleration

We next tested the association of age acceleration with plasma AD biomarkers to test effects of biological aging with chronological age regressed out (Figure 4B and Tables S7–S9) and considered associations significant if we identified FDR‐significance with either clock (or both). Of the nine biomarkers tested, eight were significantly associated with age acceleration (Figure 4B and Table S7), including three P‐tau measures 181, 217, and 231, as well as Aβ42/Aβ40, Aβ40, GFAP, total‐tau, and NfL, whereas Aβ42 was nonsignificant. These findings demonstrate that biological age has an effect on *antemortem* biomarker levels beyond the contribution of chronological age. In contrast to chronological age's association with classic neurodegenerative markers (Figure 4A and Table S1), whereby higher plasma Aβ40, GFAP, and NfL levels were associated with an older biological age, the top association with age acceleration was with the AD‐specific marker, P‐tau217 (Figure 4B and Table S7), whereby higher P‐tau217 levels were associated with a faster biological age acceleration. In ROS/MAP (Table S4), all four neuropathological measures were significantly associated with age acceleration. As with biological age, we observed a trend (in EFIGA) that age acceleration associations with biomarkers were more strongly associated in *APOE*‐ε4 non‐carriers as compared to ε4 carriers (Table S9), and we likewise observed this pattern in ROS/MAP with the *postmortem* neuropathology measures (Table S6).

#### 3.5.3 Sensitivity analysis adjusting for genetic context

We next tested the association of biological aging with biomarkers, adjusted for global genetic ancestry (see the Methods section for more details). Please note that due to the sample overlap between genetic and methylation data availability in EFIGA, the sample size for this sensitivity analysis was *N* = 588 as compared to *N* = 704 for the main analysis. Ancestry proportions were not associated with biological age or age acceleration (Table S10). All biomarkers significant from the main analysis remained significant in at least one clock (Table S11). Likewise, for age acceleration, all biomarkers significant from the main analysis remained significant in at least one clock (Table S11); to note, the top association with age acceleration remained as P‐tau217 (as mentioned in the section above).

### 3.6 Chronological aging is associated with immune cell types

We deconvoluted whole‐genome methylation data (see Methods) to determine the cell‐type proportional methylation levels in each participant. We then tested the association of each cell‐type proportion with chronological age (Figure 5 and Table S12). Of the 12 cell‐type proportions identified, seven were significant after adjusting for multiple comparisons, and the top three associations included CD8 memory T‐cells (*β* = 0.13, *p* = 1.12 × 10^−3^, p.FDR = 8.29 × 10^−3^), monocytes (*β* = 0.12, *p* = 2.07 × 10^−3^, p.FDR = 8.29 × 10^−3^), and naïve CD8 T‐cells (*β* = −0.12, *p* = 1.55 × 10^−3^, p.FDR = 8.29 × 10^−3^).

### 3.7 Biological aging is associated with immune cell types

#### 3.7.1 Biological age and age acceleration

We assessed whether biological age (Tables S12–S14) and age acceleration (Tables S15–S17) were associated with cell‐type proportions derived from deconvolution of the epigenetic data. Three cell types were significantly associated with biological age (Figure 5 and Table S12), including CD4 memory T‐cells (Horvath: *β* = −0.13, *p* = 1.00 × 10^−3^, p.FDR = 1.20 × 10^−2^), memory B‐cells (Horvath: *β* = 0.10, *p* = 7.04 × 10^−3^, p.FDR = 4.23 × 10^−2^), and naïve CD8 T‐cells (Hannum: *β* = −0.12, *p* = 1.43 × 10^−3^, p.FDR = 1.71 × 10^−2^). Additionally, the same three cell types were significantly associated with age acceleration (Figure 5 and Table S15).

#### 3.7.2 Sensitivity analysis adjusting for genetic context

Sensitivity analyses (*N* = 571 with deconvoluted methylation data and genetic data allowing for admixture calculations) yielded a slightly different set of results (Table S18). CD4 memory T‐cells remained significant in the Horvath clock age acceleration (*β* = −0.10, *p* = 8.16 × 10^−3^, p.FDR = 4.90 × 10^−2^), but natural killer (NK) cells surfaced as significantly associated with age acceleration in the Horvath clock (*β* = 0.12 *p* = 3.99 × 10^−3^, p.FDR = 4.79 × 10^−2^). No associations remained significant with biological age, but the association with NK cells was nominally significant (*p* = 5.42 × 10^−2^).

## 4 DISCUSSION

We conducted a comprehensive analysis of biological aging in Hispanics by building two established blood‐based epigenetic clocks of aging and testing associations with *antemortem* AD biomarkers, as well as validating these findings in the ROS/MAP cohort with *postmortem* brain measures. First, epigenetic age was correlated with chronological age in Hispanics, but these correlations were slightly weaker compared to non‐Hispanic whites in ROS/MAP, and this finding aligns with previously published work.^13^, ^15^, ^16^ Next, we found that biological age acceleration was significantly associated with sex, clinical diagnosis, and *antemortem* AD biomarker levels. The biomarker associations were slightly stronger among *APOE*‐ε4 non‐carriers compared to *APOE*‐ε4 carriers. Further, biological age acceleration is related to CD4 and CD8 T‐cell proportions. Our study extends prior work on epigenetic clocks by demonstrating that biological aging can predict AD risk *antemortem* in a diverse sample that is enriched for preclinical AD.

### 4.1 Biological age acceleration as a preclinical AD biomarker

The key finding of this study is that age acceleration is associated with clinical AD risk and AD plasma biomarker levels. Furthermore, accelerated biological aging correlated with elevated plasma P‐tau217 concentrations, a clinically significant AD biomarker,^38^ In contrast, chronological age showed stronger associations with increased levels of general neurodegeneration plasma markers. The effect of chronological age on neurodegenerative marker levels was stronger than the effect of biological age acceleration on P‐tau217 levels, likely reflecting the composition of our study population. Our sample is enriched for preclinical individuals with a family history of AD and those diagnosed with clinical AD, suggesting the presence of mixed pathological processes. The general neurodegenerative markers appear to capture this heterogeneous pathology, whereas P‐tau217, being AD‐specific, may more precisely identify clinically relevant associations among just those at higher risk for AD pathology, specifically. Prior literature about blood‐based epigenetic clocks’ association with dementia risk has been inconsistent, with some studies showing unsuccessful AD risk prediction,^39^, ^40^, ^41^ but other studies reporting that biological aging was associated with increased AD risk and correlated with brain measures.^7^, ^12^ Our study helps to clarify this literature, showing that biological age acceleration is significantly associated with biomarkers in blood, and we successfully validated these associations with *postmortem* AD neuropathology measures. Taken together, our findings show that biological aging can help stratify clinical and pathological AD risk, conducted fully *antemortem* and in a more noninvasive manner than with lumbar puncture. This finding has implications for the possibility that blood‐based epigenetic clocks could have future clinical utility.

### 4.2 Biological aging plays a role in AD pathogenesis of *APOE*‐ε4 non‐carriers

We explored the relationship of biological aging with AD plasma biomarkers stratified by *APOE*‐ε4 carrier status. Biomarker levels were more strongly associated with biological age and age acceleration in ε4 non‐carriers compared to ε4 carriers. Thus, biological aging may be one biochemical pathway that influences *APOE*‐ε4 non‐carriers’ risk for AD neuropathologic burden,^42^ whereas neuropathologic burden is already high in *APOE*‐ε4 carriers, irrespective, possibly resulting in methylation as a less relevant biochemical pathway to AD pathogenesis in carriers. Future work should evaluate specific DNA methylation site changes in *APOE*‐ε4 non‐carriers that may relate to AD risk and neuropathological burden specifically among this group.

### 4.3 T‐cell composition is relevant to biological age acceleration

Remodeling of T‐cell composition^43^ is a hallmark of chronological aging, including decreases in naïve CD4 and CD8 T‐cells and an increase in CD8 memory T‐cells,^44^, ^45^ which we also observed in our study. When evaluating biological aging specifically, we observed a significant decrease in naïve CD8 T‐cells and memory CD4 T‐cells, but an increase in memory B‐cells, given older biological age and faster age acceleration. This confirms prior evidence that slower biological age acceleration or a younger biological age,^46^ is associated with higher naïve CD4 and CD8 T‐cell composition.^47^, ^48^ Additionally, a recent study in a diverse cohort identified that “immunological age” was associated with entorhinal cortical thickness, an early region of change in AD, further emphasizing the importance of our findings in relation to AD risk.^49^ Our study, consistent with previous findings, may support a possible link between immunosenescence and AD risk, alluding to a phase of T‐cell remodeling in early AD that exacerbates the effect of AD pathology in early stages of disease^50^; the mechanistic link between immune cell aging and AD risk needs to be clarified in future work. Our sex‐stratified follow‐up analysis showed that immune cell‐type proportions may be more relevant to biological aging in men as compared to women, with a few associations coming close to statistical significance in men, albeit none surpassing significance, possibly due to the smaller sample size of men than women in our study. One reason for this finding could be that most women in our study were post‐menopausal, and menopause is considered an “inflection point” for immune system remodeling in women.^51^ Possibly T‐ and B‐cell composition was already lower in women^51^ in our study, and thus provided no evidence of change, whereas in men, this immune system change was more detectable. Future studies will need to disentangle the relationship between sex, immunity, menopause, and biological aging.

### 4.4 Strengths and limitations

Strengths of this study include that we conducted this entire analysis *antemortem*, noninvasively, and in a diverse sample. Our sample is enriched for those in preclinical disease, allowing for a better understanding of AD risk prior to disease onset. This study is not without limitations. First, we chose two of the most well‐established blood‐based clocks, but we did not include newer clocks, such as the cortical clock that was primarily developed for brain methylation levels (not blood) and the DunedinPACE clock, optimized for younger adults and vulnerable to survival bias of aging. As shown in other studies, biological age associations with chronological age are somewhat attenuated in non‐European ancestry groups, possibly because the clocks are constructed and trained predominantly in non‐Hispanic whites. In addition, our study design was cross‐sectional due to data availability in the EFIGA cohort, which limited our ability to make statements of predictive power of biological aging measures in relation to such longitudinal measures as incident AD dementia risk and cognitive decline. Our sample sizes of men/women and *APOE*‐ε4 carriers/non‐carriers were imbalanced, which represents the general AD population but may have affected statistical power in the smaller groups. Furthermore, cell‐type composition relevant to aging in the periphery may differ from the composition relevant in brain aging, and cell types that did not have reliable markers for deconvolution may likewise be relevant to biological aging but could not be included in this study. Additionally, because our study evaluates composite measures of biological aging consisting of a few hundred CpG sites, we are unable to link site‐specific methylation changes to gene pathways through such means as an enrichment analysis.

## 5 CONCLUSIONS

We have highlighted that plasma AD biomarker levels, directly related to AD neuropathologic burden, are associated with biological age acceleration in Hispanics, and that DNA methylation changes may be a relevant pathway for AD pathogenesis in *APOE*‐ε4 non‐carriers. We also show that a key molecular pathway in biological age acceleration is “immunological aging,” specifically, T‐cell composition changes. Our findings thus provide evidence for the future clinical utility of biological aging measures as an additional means for stratifying AD risk, while an individual is in preclinical disease stages.

## CONFLICT OF INTEREST STATEMENT

The authors do not have any conflicts of interest with the research presented in this investigation. Author disclosures are available in the Supporting Information.

## CONSENT STATEMENT

All participants of the EFIGA study provided informed consent and all secondary analyses were approved by the Columbia University Medical Center Institutional Review Board. Both ROS and MAP were approved by an Institutional Review Board of the Rush University Medical Center. All ROS and MAP participants signed an Anatomic Gift Act and informed and repository consents.

## Supporting information



Supporting Information

Supporting Information
